# Help-Seeking for Mental Health Issues in Professional Rugby League Players

**DOI:** 10.3389/fpsyg.2020.570690

**Published:** 2020-09-24

**Authors:** Susanna Kola-Palmer, Kiara Lewis, Alison Rodriguez, Derrol Kola-Palmer

**Affiliations:** ^1^Department of Psychology, University of Huddersfield, Huddersfield, United Kingdom; ^2^Department of Allied Health Professions, Sport and Exercise, University of Huddersfield, Huddersfield, United Kingdom; ^3^School of Healthcare, University of Leeds, Leeds, United Kingdom

**Keywords:** help-seeking, mental health, elite athletes, rugby football players, player welfare

## Abstract

**Background:**

Despite the prevalence and negative consequences of mental health issues among elite athletes, studies suggest many do not seek professional help. Understanding barriers and facilitators to help-seeking is imperative to reduce the burden of mental health symptoms and disorders.

**Aims:**

This study aimed to elucidate factors associated with actual help-seeking behavior in professional rugby football league (RFL) players in England and one club in France.

**Design:**

A mixed-methods cross-sectional online survey design was used, and completed by 167 professional RFL players.

**Methods:**

The survey consisted of measures of mental health, perceived psychological stress, athletic identity, knowledge of player welfare, and actual help-seeking. Additionally, one open-ended question allowed free-text comments about barriers to help-seeking.

**Results:**

Those who had sought help reported significantly higher psychological stress compared to those who had not sought help. Help-seeking was associated with better mental health literacy and higher perceived psychological stress in a multivariate analysis. The qualitative analysis revealed a number of perceived barriers to help-seeking, of which lower mental health literacy and stigma were the most prominent.

**Conclusion:**

In one of the first studies to examine actual help-seeking behavior, professional rugby players who had sought help for mental health and personal issues were characterized by having greater mental health literacy and experiencing greater psychological stress. Players also identified feelings of embarrassment, pride, fear and shame act as barriers to seeking help for mental health issues. These results suggest focusing on increasing mental health literacy and reducing stigma may increase help-seeking behavior for mental health symptoms.

## Introduction

An increasing number of studies have investigated the prevalence and treatment of mental health disorders and symptoms in elite athletes (Rice et al., 2016; Reardon et al., 2019). This led to the International Olympic Committee (IOC) calling together experts in the field to draw together a consensus statement on the management of mental health disorders in elite athletes (Reardon et al., 2019). It is recognized that throughout their careers elite athletes will be subject to both career-specific and general factors that may lead to common mental health disorders. A recent meta-analysis suggests the prevalence of anxiety and depression symptoms in current elite athletes is 34% (Gouttebarge et al., 2019), higher than prevalence estimates in the general population (15.7%; McManus et al., 2016). If untreated, CMDs can produce significant physical, psychological, social, functional and occupational disability and even premature death (Institute of Medicine, 2001; Ferrari et al., 2013; Zivin et al., 2015). Therefore, seeking professional help is imperative for the prevention, early detection and treatment of, and recovery from mental health disorders (Gulliver et al., 2012a).

However, despite this range of negative outcomes, many people who experience a CMD do not seek treatment (Jorm et al., 2017). There is evidence of less help-seeking for mental health difficulties among athletes relative to non-athletes. For example, Watson (2005) found student athletes had significantly less positive attitudes to help-seeking than their non-athlete student peers. The typical age range of elite athletes is 16–34 (Wood et al., 2017) which is also the group least likely to access support for CMDs in the general population. Also, in line with trends in the general population this is particularly so for male athletes (Watson, 2005; Lubian et al., 2016). Whilst there is increasing research on the management of mental disorders there is less research on prevention of mental health disorders or the role of early detection and intervention (Purcell et al., 2019).

Understanding barriers to help-seeking is important to reduce the burden of CMDs, and improve both clinical and social outcomes. For elite athletes a number of individual barriers to accessing help for mental health issues have been identified. This include lack of knowledge and awareness of mental health difficulties (i.e., low mental health literacy), perceived stigma, negative past experiences of help-seeking (Gulliver et al., 2012a; Castaldelli-Maia et al., 2019), and more negative attitudes toward help-seeking (Purcell et al., 2019). Further, cultural factors (such as attitudes of coaches, normalization of help-seeking) also play a role, particularly where a sport emphasizes mental and physical toughness (Baum, 2005). Evidence suggests that athletes engaged in contact sports are less likely to seek help for mental health issues compared with non-contact sport athletes (Martin, 2005). Masculine norms embodied in contact sports, e.g., competition, aggression, and toughness may lead athletes to minimize signs of weakness and limit their willingness to open up about mental health issues (Reardon and Factor, 2010). Finally, there are local cultural issues such as the extent to which an athlete’s particular sporting organization recognizes the prevalence and significance of mental health issues and attempts to develop a culture of perceived support and acceptability of help seeking (Reardon and Factor, 2010; Rice et al., 2016; Sebbens et al., 2016).

Methodologically, this previous research has focused on attitudes to or beliefs about help seeking or intentions to help seek. As such the primary outcome variable in previous research is attitudes or intentions to help seeking, rather than *actual* help-seeking behavior. While such approaches are valuable in identifying factors which underpin attitudes toward help-seeking and intentions to seek help, there is ample evidence that neither attitudes nor intentions are strong predictors of behavior (e.g., Sheeran and Webb, 2016).

Consequently, there is a lack of studies investigating influences on actual help seeking in elite athletes. The extent to which attitudes to and knowledge of mental health support predict actual help-seeking in elite athletes remain to be assessed. Most previous studies have assessed anticipated barriers to help-seeking, and none have used actual help-seeking as an outcome variable. Thus, conclusions about the extent to which previously identified barriers hinder or prevent actual help seeking should be drawn with caution.

Building on our previous work, this research sought to expand on the knowledge and understanding of professional rugby football league (RFL) players’ actual help-seeking behaviors. Players were recruited from the RFL Super League which is the top-level professional rugby league club competition in the Northern Hemisphere. We define elite athletes in line with others as those competing in the highest competitive league (Heffernan et al., 2015), and use the terms “professional” and “elite” interchangeably when referring to our sample.

In this context, it should be noted that the RFL has pro-actively sought to address mental health issues by working in partnership with Sporting Chance, a charity aimed at providing treatment and support for elite athletes in the United Kingdom. It consists of a national network of counselors and therapists offering talking therapies for mental health issues, clinics for treating addictive disorders (gambling, alcohol, drugs), and an education department delivering seminars to staff and players at clubs and governing bodies across sport in the United Kingdom. It is estimated over 400 RFL players have been helped by Sporting Chance since 2011 (Rugby League, 2018). It should be noted some clubs also employ a chaplain who may also have been a source of support for some players seeking help for their mental health, and players may have sought help through access to medical staff in their respective countries. Identifying the correlates of actual help seeking is of importance for those actively engaged in elite sport, from elite athletes to coaches and support staff, to governing bodies. The results can inform interventions, future research and policy, mental health education and support, within rugby football league and the wider sporting context. We aimed to identify and elucidate possible correlates of *actual* help-seeking for mental health issues in professional RFL players in England and the one club in France.

## Materials and Methods

### Design

This is a secondary data analysis of a larger project and the design has been described elsewhere (Kola-Palmer et al., 2019). Briefly, a cross-sectional online survey was conducted between January and March 2015 and again between January and March 2016. The survey consisted of psychometric measures of psychological stress, mental health, athletic identity, as well as our own constructed player welfare questionnaire, and finally an open-ended question about barriers to help-seeking to elicit qualitative responses. Responses were sought from players in the Super League rugby clubs in England, including the one club based in France. Participant inclusion criteria included being a professional rugby player and currently playing for one of the Super League Rugby clubs. All procedures were reviewed and approved by the host institutions’ ethics panel. The results presented here represent the data from survey 2.

### Participants

The participants were professional rugby players recruited from all Super League clubs in the United Kingdom and the one club based in France. Responses were completed from 167 professional rugby players (mean age = 24.89, *SD* = 4.62), providing a response rate of approximately 45%. The participants had played full time rugby league for an average of 6.12 years (*SD* = 4.74), and 151 players (73.3%) played for the same club the previous season.

### Measures

#### Mental Health Scale

The five-item Mental Health Index (MHI-5) of the 36-item Short Form health survey (SF-36) (Ware and Sherbourne, 1992) was used to assess current general mental health problems. It is a brief screening questionnaire for depression and anxiety disorders, and is a simple and valid tool, with good specificity and sensitivity for detecting mood disorders in the general population (Rumpf et al., 2001; Cuijpers et al., 2009). It requires respondents to consider events in the past month (e.g., “how much of the time in the previous 4 weeks have you been a very nervous person”), and responses are on a six-point scale from 1 (all of the time) to 6 (none of the time). After coding, adding, and transforming MHI-5 scores range from 0 (worst) to 100 (optimal mental health). Psychometric properties have been established (Rumpf et al., 2001). For the current sample, good internal consistency was found, and Cronbach’s alpha was 0.76.

#### Psychological Stress Scale

The 10-item Perceived Stress Scale (PSS-10) was used to assess the extent to which players perceived situations in life as stressful (Cohen et al., 1983; Cohen and Williamson, 1988). Items tap how unpredictable, uncontrollable, and overloading respondents find their lives, and respondents were asked to reflect on events over the past month (e.g., “In the last month, how often have you felt that you were on top of things”). The responses are on a 5-point scale ranging from 0 (never) to 4 (very often), and a higher score indicates greater psychological stress. Validity and reliability have been established (Cohen et al., 1983; Lee, 2012). Excellent internal consistency was found for the present sample, and Cronbach’s alpha was 0.81.

#### Athletic Identity

The Athletic Identity Measurement Scale (AIMS; Brewer and Cornelius, 2001) was used to assess the extent to which players identify with the athlete role. The seven-item scale requires respondents to indicate the extent to which they agree to statements relating to aspects of identification (e.g., social identity, exclusivity, and negative affectivity) with the athlete role. The responses are on a scale from 1 (strongly disagree) to 7 (strongly agree), and higher scores indicate stronger identification with the athlete role. Sound psychometric properties have been established (Brewer and Cornelius, 2001; Visek et al., 2008). For the current sample, Cronbach’s alpha was 0.76, indicating good internal consistency.

#### Player Welfare Questionnaire

A survey consisting of five subsections, relating to knowledge of and attitudes to RFL club welfare managers and policies (seven items, Cronbach’s alpha 0.93), financial advice and education (four items, Cronbach’s alpha 0.91), mental health supports (seven items, Cronbach’s alpha 0.87), life style issues (i.e., gambling, addictions; 12 items, Cronbach’s alpha 0.90), and life after rugby (i.e., career transition, education; four items, Cronbach’s alpha.90) was constructed. Respondents were asked to rate each statement (e.g., “My club has a welfare manager,” “I know how to access counseling, Sporting Chance and other mental health services should I need to”) on a 5-point Likert scale from 1 (strongly disagree) to 5 (strongly agree), and a higher score indicates higher agreement.

#### Help-Seeking

Help-seeking was assessed by one item. Respondents were asked “Have you accessed Sporting Chance?” with a “yes” or “no” response option.

#### Open-Ended Questions

Respondents were asked “In your opinion, what are the main reasons that prevent players from accessing player welfare supports?” and were provided with an essay style box for comments.

### Procedure

Participants were recruited from across all Super League rugby clubs which at the time were mainly based in England, with one French club. The players were made aware of the survey link by each Super League Rugby club’s player welfare manager, who invited all first team players to take part in the survey. Players completed the anonymous, self-administered 20 min online survey in their own time in self-selected locations. The survey was made available for a period of 6 weeks, with regular reminders from the player welfare manager.

### Statistical Analyses

To examine the relationship between selected variables and actual help-seeking behavior a logistic regression analysis was used. Decisions to include variables were based on known clinical and theoretical importance and univariate analysis of each variable (Bursac et al., 2008). The variables selected for inclusion were psychological stress scores, MHI-5 scores, athletic identity scores, and the scores on the welfare policies, mental health support, lifestyle policies, along with the demographic variables marital status, and children. Marital status and number of children were collapsed into binary variables (married vs. single, and children/no children).

### Content Analysis

The open-ended questions were analyzed using content analysis, as it allows for the identification, analysis and reporting of patterns within non-numerical data (Griffiths, 2016). Given the limited nature of responses, we undertook thematic coding, rather than a full thematic analysis. The thematic coding was conducted by three researchers involved in the study. Categorisation of items were completed through the use of initial codes, and subsequently linked into major themes. Disagreements were resolved through discussions.

## Results

### Descriptive Statistics and Preliminary Analyses

Across the respondents, 26 players (15.6% of the sample) had sought help from Sporting Chance. Players who had sought help were similar to those who had not on a number of variables, including age, duration of play, mental health score, and athletic identity, all *p*s > 0.05. There was, however, a statistically significant difference in perceived stress, with those who had sought help reporting higher stress scores (*M* = 16.50, *SD* = 6.44) than those who had not sought help (*M* = 13.61, *SD* = 5.36), *t*(165) = 2.35, *p* = 0.02.

There was an association between help-seeking and knowing others who have sought help χ^2^(1) = 4.31, *p* = 0.038. Players who had sought help themselves were more likely to know of others who had sought help. Please see Table 1 for the demographic information.

**TABLE 1:** Demographic information broken down by help-seeking.

	Have sought help (*n* = 26)		Have never sought help (*n* = 141)	
Variable	Mean (*SD*)	Frequency (%)	Mean (SD)	Frequency (%)
Age	23.92 (3.66)		25.11 (4.76)	
Years of professional rugby experience	5.54 (3.49)		6.15 (4.48)	
Marital status				
Married/Living as married		6 (23.1)		57 (40.4)
Single		18 (69.2)		81 (57.4)
Divorced/separated		1 (3.8)		1 (.7)
Have children				
No		20 (76.9)		90 (63.8)
Yes		6 (23.1)		51 (36.2)
Know of others having accessed Sporting Chance				
No		8 (30.8)		70 (49.6)
Yes		16 (61.5)		54 (38.3)
Missing		2 (7.7)		17 (12.1)
MHI score	69.69 (16.40)		75.21 (15.64)	
PSS score	16.50 (6.44)		13.61 (5.63)	
Athletic identity	39.58 (5.29)		37.09 (6.30)	
Welfare policy	37.65 (9.51)		37.95 (6.71)	
Financial education	14.35 (4.36)		13.68 (3.77)	
Mental health supports	28.46 (3.86)		26.04 (5.27)	
Lifestyle supports	48.35 (8.90)		45.77 (8.41)	
Life after rugby	15.15 (3.73)		14.92 (3.39)	

### Correlates of Help-Seeking

Direct logistic regression was performed to assess the impact of a number of factors on the likelihood of professional rugby players seeking help. The model contained eight independent variables (psychological stress, symptoms of depression and anxiety, athletic identity, welfare policies, mental health support, lifestyle policies, marital status, children). The full model containing all predictors was statistically significant, χ^2^(*df* = 8) = 20.61, *p* = 0.009, indicating the model was able to distinguish between respondents who reported help-seeking and those who did not report help-seeking. The model as a whole explained between 11.8% (Cox and Snell *R*^2^) and 20.6% (Nagelkerke *R*^2^) of the variance in help-seeking, and correctly classified 86% of the cases. As shown in Table 2, two of the independent variables made a unique statistically significant contribution to the model. The strongest predictor of reporting help-seeking was mental health literacy, recording an odds ratio of 1.22. This indicated that respondents who had better awareness of mental health support were 1.22 times more likely to report help-seeking than those who had less awareness of mental health support, controlling for all other factors in the model. Additionally, those with higher levels of perceived stress were 1.16 times more likely to have sought help than those who reported lower perceived stress.

**TABLE 2:** Logistic regression model estimating effects of variables on help-seeking.

Variable	*B*	*SE*	*p*-value	*OR*	95% CI
Stress	0.15	0.06	0.021	1.16*	1.02–1.31
Mental health	0.01	0.02	0.486	1.01	0.98–1.06
Athletic identity	0.03	0.05	0.495	1.03	0.95–1.13
Welfare policies	−0.05	0.04	0.193	0.96	0.89–1.02
MH supports	0.20	0.08	0.013	1.22*	1.04–1.43
Lifestyle	−0.02	0.04	0.711	0.99	0.91–1.07
Marital status	0.60	0.62	0.331	1.86	0.55–6.06
Children	0.31	0.65	0.636	1.36	0.38–4.87
(Constant)	−10.69	3.54	0.003		

Model χ^2^ = 20.61, df = 8, p < 0.01, *p < 0.05.

### Content Analysis

Of the 167 respondents to the survey, 95 (57%) provided comments to the open-ended question about barriers to help-seeking. There was no association between providing comments to this question and help-seeking behavior [χ^2^(1) = 0.08, *p* = 0.785], marital status [χ^2^(1) = 0.03, *p* = 0.84], or having children [χ^2^(1) = 1.13, *p* = 0.288]. Similarly, there were no statistically significant differences between those who provided comments and those who did not on age, mental health score, perceived stress score, athletic identity, awareness of welfare policies, or awareness of financial education, all *p*s > 0.05. Those who provided answers had, however, significantly higher scores on awareness of mental health supports [*t*(166) = 2.08, *p* = 0.039], and lifestyle policies [*t*(166) = 2.34, *p* = 0.020] than those who did not.

Answers to question “In your opinion, what are the main reasons that prevent players from accessing player welfare support?” were then thematically coded enabling a number of barriers to help-seeking being identified. These were poor mental health literacy, perceived stigma, personal characteristics and attitudes, and instrumental barriers.

The poor mental health literacy theme comprised player perceived lack of knowledge and awareness of mental health problems and treatment, including not knowing where to seek help and thinking they don’t need help. Perceived stigma included the subthemes of embarrassment, pride, fear of weakness, and shame. Personal characteristics and attitudes comprised the subthemes players own decision, lack of interest, and lack of confidence/trust in system. The major theme instrumental barriers included the subthemes lack of time, recentness of player welfare and lack of communication. Additionally, a number of respondents answered “don’t know” to this question. See Table 3 for a summary of the thematic coding.

**TABLE 3:** Summary of content analysis of the open-ended question.

Major Theme	Subthemes	Frequencies	Examples
Poor mental health literacy	Lack of knowledge, incl. Not knowing where to seek help Don’t think they need help	32/100	“Not knowing who to get in contact with about certain questions or problems” “Lack of understanding of what help can be received” “Some players think they don’t need support” “We don’t realize we need it”
Perceived Stigma	Embarrassment Pride Fear Shame	30/100	“Probably too embarrassed to come forward with their problem” “Feel too proud” “Worried about looking soft” “They feel it’s a sign of weakness to talk to someone about their problems” “Shame of asking for help”
Personal characteristics and attitudes	Players own decision Lack of interest Lack of confidence/trust in system	16/100	“Themselves not wanting to ask for help” “Own decisions” “Not bothering with it” “No trust in the system” “Trust to talk about problems”
Don’t know/Unsure		12/100	“Not sure why they wouldn’t” “At my club there isn’t any reason, as the welfare supporters are very good” “I don’t know”
Practical/Instrumental barriers	Lack of time Recency of player welfare Lack of communication	10/100	“Reluctance to stay at training for a longer period to discuss things with welfare manager” “Wasn’t in the past as accessible” “Lack of communication”

Although there was no difference in actual help-seeking between players who did or did not answer the open-ended question, the players who provided answers about barriers to help-seeking had significantly better mental health literacy than players who did not answer this question. It is therefore interesting that what emerges from the content analysis is that poor mental health literacy and perceived stigma are the two most frequently mentioned barriers to help-seeking among elite rugby football league players. The players themselves are citing lack of knowledge and awareness of where to seek help as a major barrier to help-seeking, along with the perceived stigma of appearing “weak.” The content analysis thus supports the regression analysis, and highlight the importance of mental health literacy and awareness of available help and how to access it.

## Discussion

This study provides, to the best of our knowledge, the first analysis of factors associated with actual help-seeking for mental health issues in elite athletes. The main results can be summarized as follows. First, mental health literacy was a significant and independent correlate of actual help-seeking behavior, after controlling for other variables. Players who had better mental health literacy were more likely to have sought help than players with less knowledge of mental health support, independent of other factors. Second, psychological stress was a significant and independent correlate of actual help-seeking behavior. Players who reported more psychological stress were more likely to have sought help than players who reported less psychological stress, independent of other factors. Finally, when asked to reflect on barriers to help seeking, players reported that poor mental health literacy and perceived stigma are the leading perceived barriers, followed by personal characteristics and attitudes, including concerns of confidentiality and trust.

To the best of our knowledge, this is the first study to have assessed predictors of actual help-seeking behavior in elite athletes. RFL players who had sought help were characterized by having greater mental health literacy and experiencing greater psychological stress. Conversely, non-help seeking was characterized by less knowledge of available mental health support (lower mental health literacy) and lower perceived stress levels. The lower perceived stress levels reported by these players may suggest lower need for help-seeking, but the lower help-seeking could also reflect having less knowledge of where to seek help. Help-seeking is an important step toward accessing appropriate mental health support and improving clinical and social outcomes. In order to seek out help for mental health issues, someone must first recognize there is a problem, make the decision to seek help, and select an appropriate service (Cauce et al., 2002). Mental health literacy underpins problem recognition, which is the first step in realizing help may be needed and that help is available. Mental health literacy is multifactorial and includes knowledge of prevention of mental health issues, recognition of developing mental health issues, knowledge of help-seeking options and available treatments, and first aid skills to support others who are at risk of developing mental health issues (Jorm, 2012).

These results suggest that to improve the mental health wellbeing of elite athletes, focus should be on improving mental health literacy. This includes awareness of available support, and how to access it, as well as ability to recognize signs and symptoms of common mental health disorders (CMDs). There has been a growing recognition that mental health literacy is an important component of elite athlete welfare. For example, in our previous work we found that better mental health literacy was a significant and independent correlate of wellbeing in elite RFL players (Kola-Palmer et al., 2019). A number of research studies have evaluated interventions developed to influence mental health literacy. The mental health literacy workshop for elite sports coaches and support staff developed by Sebbens et al. (2016), as mentioned above, successfully increased knowledge of CMDs and improved participants’ confidence to help someone with a mental health difficulty. This is useful for increased recognition of mental health issues in elite athletes by coaching and support staff. The authors suggest that even a brief intervention can aid in promoting early intervention and timely referral of elite athletes with mental health concerns to appropriate professionals. However, no measure of actual behavior change was included, which warrants future research to explore behavioral outcomes. Additionally, the Sebbens et al.’s (2012) study was not focused on increasing the mental health literacy of elite athletes themselves. Studies which have focused on improving mental health literacy in elite athletes have suffered from low sample sizes, and so further research is required.

A recent study of college students found that mental health literacy and perceived stigma had significant and unique independent effects on help-seeking attitudes (Cheng et al., 2018). The authors conclude that these should be considered concurrently when conceptualizing help-seeking attitudes. Our study extends this knowledge to include elite rugby football league players. In the free-text responses, players talked about how feelings of embarrassment, pride, fear, and shame act as barriers to seeking help for mental health issues. In the context of the sporting world, mental toughness is highly valued, and elite athletes are expected to not show signs of weakness (e.g., Lebrun et al., 2018). When elite athletes consider mental health difficulties as a sign of weakness, incompetence or personal flaw, they are likely to experience shame in response to mental health issues. This supports the idea that it is neither expected nor accepted for elite male athletes to display any signs of weakness (Doherty et al., 2016). This arguably contributes to the denial or hiding of mental health issues for fear of the potential consequences of such a disclosure on the elite athlete career (e.g., Hill et al., 2016; Uphill et al., 2016; Lebrun et al., 2018). Help-seeking is a process, whereby someone first must become aware of the problem, and perceive a need for help, then identify appropriate sources for help to access, and finally be willing to seek out and disclose their issues to the source of help (Cauce et al., 2002). Thus, it is unlikely that solely focusing on increasing mental health literacy, without considering reducing stigma will foster help-seeking in RFL players. Our finding that players who had sought help themselves were more likely to know of other players who had sought help, is reflected in our study with RFL Player Welfare Managers (PWM; Lewis et al., 2018). In interviews with PWMs, a snowball effect was revealed, in which one player asking for help leads to another asking for help. This suggests that changing the culture to allow elite athletes to be more open may facilitate help-seeking. Coaching staff have an important role to play in ensuring a supportive culture in which help-seeking is encouraged (Castaldelli-Maia et al., 2019).

Additionally, players perceived barriers to help-seeking included lack of confidence or trust in the support system. This supports studies which have found that having established and trusted relationships with service providers facilitates help-seeking (Rickwood et al., 2007; Vogel et al., 2007; Gulliver et al., 2012a). Confidentiality is necessary to build trust between the health professional and the client, however, in the elite sporting world, information about an athlete is often freely passed between medical staff and coaches for the good of the athlete (López and Levy, 2013). Elite athletes may fear that opening up about mental health symptoms or difficulties would negatively impact their chances of a successful athletic career (e.g., being excluded from the team, not signing a new contract or an advertising campaign). Athletes may also fear negative reactions from their teammates and coaches if they open up about mental health symptoms or difficulties (Castaldelli-Maia et al., 2019). Confidentiality, privacy and trust must be promoted with elite athletes and particularly with coaching and support staff, because they may expect to be kept informed about their athletes. Additionally, anti-stigma interventions may serve to change attitudes toward help-seeking in elite athletes as well as coaching and support staff. Such interventions have been found to reduce stigma around depression and psychosis (Bapat et al., 2009), and anxiety (Gulliver et al., 2012b). The extent to which anti-stigma interventions influence actual help-seeking and access to care remain to be established.

The open-ended question also uncovered practical barriers to help-seeking, such as busy schedules and lack of time. This is in line with previous research which found that lack of time was a chief barrier to help-seeking in college athletes (López and Levy, 2013). Together this highlights the importance of making appropriate services and sources of help available and accessible to athletes taking into account their demanding schedules, time constraints and needs (López and Levy, 2013; Castaldelli-Maia et al., 2019).

### Limitations

The following limitations should be noted. First, this study used a cross-sectional design, so no causal inferences can be made. We have identified a number of correlates of actual help-seeking in elite athletes, which warrant further longitudinal research to establish predictive power. Second, given the voluntary nature of the survey, response bias cannot be ruled out, which could limit the generalizability of our findings. We aimed to recruit from all RFL Super League Teams, giving us a potential participant pool of approximately 372 contracted elite players. However, we have no way of knowing if all players were made aware of the survey. Nonetheless, the sample characteristics closely match that of the entire population of RFL players on the basis of age and playing experience. The study findings are also in line with and extend those of previous research studies, giving us a degree of confidence in our interpretations. Third, help-seeking was assessed by just one item, and related to only one source of help (Sporting Chance). Thus, we have no way of knowing if players had sought help for mental health symptoms from other services or sources (e.g., GP, club chaplain) and thus underestimated help-seeking. Future studies should include a range of sources of help to gain a more comprehensive picture of help-seeking in elite athletes. Finally, in this paper we analyzed data that were available to us. Future studies should include a measure of stigma, to fully explore the relationship between mental health literacy, psychological stress, stigma and actual help-seeking.

### Suggestions for Future Research

The extent to which mental toughness impacts on help-seeking in elite athletes warrants further study. The proposed link between the culture of being physically and mentally tough, and denying signs of weakness, and perceptions of stigma on actual help-seeking behavior should be further explored. Additionally, sufficiently powered intervention studies aimed at increasing mental health literacy and subsequent impact on actual help-seeking behavior are required to allow a thorough assessment of such interventions.

### Implications and Recommendations

The results from the present study suggest that actual help-seeking for mental health difficulties in elite athletes can be improved through increased mental health literacy and reduced stigma around mental health help-seeking. These are indeed global challenges (Castaldelli-Maia et al., 2019). By educating athletes, coaches and high performance directors about symptoms of different mental health conditions, mental health issues in elite athletes can be normalized, prevented and/or detected (Moesch et al., 2018). Moesch et al. (2018) further argue that normalizing and validating the issues of mental health difficulties and vulnerability in elite sports is an important step that staff and sport organizations can do to reduce the stigma in elite athlete populations. Additionally, all behaviors that will facilitate help-seeking when athletes suffer with mental health difficulties should be reinforced. There should be a clear pathway and signposting by institutions and stakeholders (knowledge about what support is available and actual access to the support). Acknowledging the time pressures and competing demands elite athletes experience is also important, to ensure help and support is available. These findings have relevance to sporting organizations, coaches, welfare officers and anyone involved in elite athlete care in reducing barriers and improving accessibility to mental health services.

## Data Availability Statement

The raw data supporting the conclusions of this article will be made available by the authors, without undue reservation, to any qualified researcher.

## Ethics Statement

The studies involving human participants were reviewed and approved by the Human and Health Sciences School Research Ethics and Integrity Committee (SREIC) University of Huddersfield. The patients/participants provided their written informed consent to participate in this study.

## Author Contributions

SK-P and AR conceived and planned the study. SK-P collected the quantitative data for T1 and T2. AR collected the qualitative data for T1. KL collected the qualitative data for Time 2. SK-P analyzed the quantitative data, while KL and DK-P analyzed the qualitative data. SK-P wrote the manuscript with critical input from DK-P. All authors contributed to the interpretation of the results and provided critical feedback and helped shape the analysis, conclusions, and manuscript.

## Conflict of Interest

The authors declare that the research was conducted in the absence of any commercial or financial relationships that could be construed as a potential conflict of interest.
